# QUALITY OF LIFE AFTER VERTICAL GASTRECTOMY EVALUATED BY THE BAROS QUESTIONNAIRE

**DOI:** 10.1590/0102-6720201700010005

**Published:** 2017

**Authors:** Giselle Abigail MENDES, Guilherme Pedroso VARGAS

**Affiliations:** 1 Department of Medicine, Regional University of Blumenau; 2 Clinic of Surgery of Digestive System - VIDAR, Blumenau, SC, Brazil

**Keywords:** Bariatric surgery, Gastrectomy, Obesity, Quality of life.

## Abstract

**Background:**

The satisfactory outcome in the surgical treatment of obesity must include, in addition to weight loss, a significant change in the pre-existing comorbidities and in the quality of life.

**Aim:**

To evaluate the quality of life in the late postoperative period in patients that underwent videolaparoscopic sleeve gastrectomy.

**Methods:**

It was applied the questionnaire "Bariatric Analysis and Reporting Outcome System" (BAROS) in patients that underwent videolaparoscopic sleeve gastrectomy.

**Results:**

A total of 47 patients between 21-60 years old were evaluated. The total mean of BMI before surgery was 43.06±5.87 kg/m². The average percentage of the reduction of excess weight after surgery was 85.46±23.6%. The score obtained by patients in the questionnaire about the improvement in the quality of life showed excellent (36.17%), very good (40.43%), good (21.28%) and reasonable (2.13%) results. There was clinical improvement after surgery in all comorbidities investigated.

**Conclusion:**

The weight loss was critical to improve the quality of life and offered the resolution or clinical improvement in all of the investigated comorbidities in patients submitted to sleeve gastrectomy.

## RESUMO

**Racional:**

O desfecho satisfatório na abordagem cirúrgica da obesidade deve contemplar, além da perda de peso, alteração significativa nas comorbidades preexistentes e na qualidade de vida dos pacientes.

**Objetivo:**

Avaliar a qualidade de vida no pós-operatório tardio de pacientes submetidos à cirurgia de gastrectomia vertical por videolaparoscopia. ***Métodos***: Foi aplicado o questionário "Bariatric Analysis and Reporting Outcome System" (BAROS) em pacientes submetidos à gastrectomia vertical por videolaparoscopia.

**Resultados:**

Foram avaliados 47 pacientes, entre 21 e 60 anos de idade. O IMC médio antes da operação era 43,06±5,87 kg/m². A média percentual de redução do excesso de peso após foi de 85,46±23,6%. A pontuação obtida pelos pacientes no questionário sobre a melhora na qualidade de vida evidenciou resultado excelente (36,17%), ótimo (40,43%), bom (21,28%) e razoável (2,13%). Houve melhora clínica após a operação em todas as comorbidades investigadas.

**Conclusão:**

A perda de peso foi fundamental para a melhoria na qualidade de vida e proporcionou resolução ou a melhora clínica em todas as comorbidades investigadas.

## INTRODUCTION

Overweight and obesity are defined as an excessive accumulation of body fat that can affect health. Obesity is considered a chronic and multifactorial disease, and it is associated with several comorbidities and severe losses in quality of life^29^. Data from the World Health Organization showed that the prevalence of obesity has more than doubled worldwide since 1980 and in 2014 about 600 million people were obese. In Brazil, more than half of the population is overweight, and 17.9% of Brazilians are already considered obese^20^. To combat this disease, clinical treatment is the first approach and includes the implementation of special diets, psychotherapy, physical activity and pharmacotherapy^20^. However, the clinical therapy for obesity, especially for severe obesity (body mass index greater than 35 kg/m²), has limited success in the short term and almost nonexistent in the long term, when compared to surgical treatment^21^.

The surgical approach for severe obesity is called bariatric surgery and appeared around 1950. Over the following years, several techniques and analysis were developed and they recognized this procedure as effective both in weight loss and in reduction of comorbidities, providing increased survival compared to clinical approaches only^5^ ^,^ ^8^ ^,^ ^27^.

Surgery is indicated for patients with Body Mass Index (BMI) greater than 40 kg/m² or greater than 35 kg/m^2^ when holding comorbid conditions that threaten life, as type 2 diabetes mellitus, hypertension, dyslipidemia, arthropathy, and others. This approach is also possible for patients with a poor clinical treatment of at least two years^11^. There are several possible surgical techniques, which basic principles are based on constraint (decrease of food intake by reducing the size or stomach capacity), malabsorption (decreased food contact time with the gastrointestinal tube) or an association of both^27^.

The procedure that patients underwent in this article is called "vertical gastrectomy" or "gastric sleeve". It is a restrictive procedure that involves the removal of the greater curvature of the stomach, starting from 4 to 6 cm from the pylorus to the esophagogastric angle, leaving the new shell with a tubular and elongated shape^6^. This technique has the advantage of not generating malabsorption problems and for not change the contact of the food with the intestinal walls and with their digestive enzymes. In addition, by not having digestive anastomoses, offers lower risk of complications in the postoperative period, when compared to other surgical techniques^3^ ^,^ ^7^ ^,^ ^17^.

However, as many factors other than weight are modified after this procedure, it is essential to evaluate the quality of life of these patients. Thus, in order to reinforce the importance of continuity of care in the postoperative period it was developed in 1998 the "Bariatric Analysis and Reporting Outcome System Protocol" (BAROS). This instrument has emerged as a simple, cheap and reliable evaluation of self-perceived quality of life in patients in the postoperative period of weight reduction surgery^23^. It is used and recognized internationally due to its practicality and efficiency to measure the results of the surgical treatment.

The BAROS protocol consists of three major areas of analysis (weight loss, medical conditions and quality of life questionnaire), which obtains a maximum score of three points for each category, totaling a maximum of nine points. The score of the category "weight loss" is given by the percentage of excess weight loss (% EWL). In "medical conditions", the individual reaches a higher score as there is clinical improvement or cure of one, several or all preexisting comorbidities before surgery. Finally, the "questionnaire of quality of life" includes questions about physical activity, social interaction, working performance, sexual interest and improvement in the general condition^25^. The sum of the three major areas generates the final score of the protocol. The result evaluates if the quality of life after surgery is worse, reasonable, good, great or excelent^23^.

Thus, the purpose of this study was to apply this protocol in patients who underwent stomach reduction surgery through laparoscopic vertical gastrectomy.

## METHODS

This article is the result of a cross-sectional study conducted between the months of April and May of 2016. The sample was selected from a clinical database of patients undergoing laparoscopic vertical gastrectomy surgery by the staff of the Clinic of Surgery of Digestive System - VIDAR, Blumenau, SC, Brazil between 2012 and 2015. The project was approved by the Ethics Committee of the Regional University of Blumenau number 1519860.

Patients were contacted and invited to participate in person filling the printed BAROS questionnaire. The same questionnaire adapted in "Google Forms" platform was sent by e-mail if impossibility of displacement to the clinic. Exclusion criteria were: a) Patients who had any cognitive limitation to compromise responses to data collection instrument; b) who refused to answer the questionnaire; c) who did not answered a question of the form. The study included only patients who were in the late postoperative period. The data were divided into three categories, according to the period elapsed after surgery: a) up to six months; b) seven to 12 months; c) more than 12 months. 

### Statistical analysis

Was performed using Epi Info software, version 7. The data level of significance was p<0.05.

## RESULTS

A total of 47 patients were evaluated, of whom 76.6% (n=36) were female and 23.4% (n=11) male. The average age was 37,3±10,75 years. The average weight and BMI before surgery were, respectively, 121.05±22.56 kg and 43,06±5.87 kg/m^2^. The average overweight of the patients was equal to 51.01±18.48 kg, an equivalent to 40.93±7.65% excess of average body weight.

All 47 patients completed the questionnaire BAROS, generating the following results: 36.17% (n=17) were classified as "excellent"; 40.43% (n=19) as "great"; 21.28% (n=10) as "good"; and 2.13% (n=1) as "reasonable" improvement of quality of life. No patient had a score in the "bad" category (Table 1).

**TABLE 1:** Final result of BAROS protocol

BAROS (points)	n	%
Bad (0-1)	0	0%
Reasonable (1-3)	1	2.13%
Good (3-5)	10	21.28%
Great (5-7)	19	40.43%
Excellent (7-9)	17	36.17%
Total	47	100.00%

There was no statistically significant difference between the score of men and women (p>0.05). The time of postoperative period was also not significant in influencing the BAROS score (p>0.05). In patients with more than six months after the surgery, it was observed predominance of excellent (71.58%) and great (76.84%) results. It was also found that the highest scores were obtained for those patients who had lost more than 75% of overweight (p<0.05).

**FIGURE 1:**
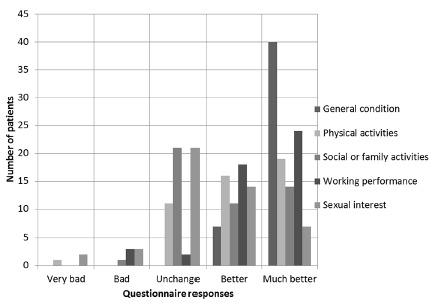
Graph with the result of BAROS by area searched

For the five domains assessed by questionnaire, the results were as follows: a) General condition: all patients responded "very good" (n=40 or 85.11%) or "good" (n=7 or 14.89%); b) Social or family activities: 21 of 47 patients (44.68%) claimed to have been no change in this area, and one patient (2.13%) reported worsening of social relationships; "improved a lot" got 29.79% (n=14) and the "improved" 23.40% (n=11); c) Physical activities: "increased a lot" 40.43% (n=19), "increased" 34.04% (n=16), "unchanged" 23.40% (n=11) and "decreased" 2.13% (n=1); d) Sexual interest: "greatly increased" 14.89% (n=7), "increased" 29.79% (n=14), "unchanged" 44.68% (n=21), "decreased" 6 38% (n=3) and "greatly diminished" 4.26% (n=2); e) Working performance: "increased a lot" 51.06% (n=24), "increased" 38.30% (n=18), "unchanged" 4.26% (n=2) and "decreased" 6.38% (n=3) (Figure 1).

The gender of the patient was not a significant factor in EWL% (p>0.05). A total of 69.4% of women lost more than 75% overweight vs. 45.4% for men. However, men lost more total weight in kilograms (72.7% lost between 40-80 kg against 38.9% of women). Patients with grade 3 obesity (BMI greater than or equal to 40 kg/m²) statistically (p<0.05) obtained better results in the loss of excess weight: 65.9% of patients lost more than 50% of the excess of weight.

The time of postoperative period exerted a significant influence (p<0.01) in reducing the BMI of patients: limited reductions, of less than 10 kg/m², were found in individuals living a postoperative period of less than six months; in contrast, 52.63% with more than 12 months lost between 15-30 kg/m².

Regarding comorbidities among patients, 55.3% (n=26) had hypertension, 15% (n=7) had type 2 diabetes mellitus, 27.6% (n=13) had dyslipidemia, 44.7% (n=21) had obstructive sleep apnea, 49% (n=23) suffered from some form of joint problem and 49% (n=23) had depressive disorder before performing the surgical procedure to reduce weight. These comorbidities were not diagnosed or classified by the authors, but self-reported by patients included in this study.

It was observed in the postoperative period that 77% (n=20) of patients who had high blood pressure achieved complete resolution of this disease and 38.4% were able to maintain control of hypertension with fewer antihypertensive medications. Among patients with type 2 diabetes mellitus, 71.4% (n=5) had complete resolution of disease and 28.5% (n=2) were able to reduce medication for glycemic control. Those who had dyslipidemia, 92.3% (n=12) showed no more such comorbidity and among those suffering from obstructive sleep apnea syndrome, 57.1% (n=12) did not report symptoms. Of individuals who had some kind of joint problem, 61% (n=14) extinguished this disease and did not use medications anymore; 34.7% (n=8) were able to reduce the number of painkillers and other medicines. Finally, of patients who had depression in preoperative period, 39.1% (n=9) reduced the number and/or concentration of antidepressant medications and 48% (n=11) did not have symptoms anymore (Table 2 and Figure 2).

**TABLE 2:** Clinical evolution of comorbidities in the postoperative considering all periods of the patients who underwent sleeve gastrectomy

Comorbidities		Total resolution	Parcial resolution	Unaltered treatment	Total
Hypertension	n	20	5	1	26
	%	77%	19,2%	3.84%	100%
Dyslipidemia	n	12	1	0	13
	%	92,3%	7,7%	0%	100%
Type 2 diabetes mellitus	n	5	2	0	7
	%	71,5%	28,5%	0%	100%
Joint problems	n	14	8	1	23
	%	61%	34,7	4.3%	100%
Obstructive sleep apnea	n	12	7	2	21
	%	57,1%	33,3%	9.5%	100%
Depressive disorder	n	11	9	3	23
	%	48%	39%	13%	100%

In relation to the average weight loss, patients who had undergone surgery within six months (n=3) lost 31.67±16.77 kg; between seven and 12 months (n=25), 38.36±8.94 kg; more than 12 months (n=19), 45.83±12.12 kg. The average reduction of BMI was 10.53±3.83 kg/m^2^, 13.73±2.8 kg/m^2^ and 16.32±3.99 kg/m^2^ and EWL% was 75.60±7.97% (n=3), 80.82±20.43% (n=25) and 93.13±27.42% (n=19), respectively.

Finally, the average weight after surgery was 80.1±17.25 kg; BMI, 28.48±4.89 kg/m^2^; overweight, 10.01±14.08 kg equivalent to 9.72±15.25%; the EWL% of all patients was 85.46±23.6% (Tables 3 and 4).

**TABLE 3:** Average weight loss, reduction of BMI and percentage of loss of excess weight per time interval of postoperative vertical gastrectomy

Time interval of postoperative	n	Average of weigh loss (kg) ±SD	Average of BMI reduction (kg/m²) ±SD	Excess weight loss % (EWL%)* ±SD	p
0 to 6 months	3	31.67±16.77	10.53±3.83	75.60±7.97	<0.01
7 to 12 months	25	38.36±8.94	13.73±2.8	80.82±20.43	<0.01
More than 12 months	19	45.83±12.12	16.32±3.99	93.13±27.42	<0.01
TOTAL	47	40.95±11.42	14.57±3.69	85.46±23.6	

%EWL= BMI before - after BMI) * 100 / (BMI before - ideal BMI)

**TABLE 4:** Characteristics of patients preoperatively surgery for weight reduction and anthropometric data pre- and post-procedure

Characteristics	Value±SD (n=47)	
Age (years)	37,3±10,75	
Height (m)	1,7±0,07	
Ideal weight (kg)	70,04±6,44	
Weight (kg)	121,05±22,56	
Overweight (kg)	51,01±18,48	
Overweight (%)	40,93±7,65	
BMI (kg/m2)	43,06±5,87	
Gender	F=36; M=11	
Comorbidities		
At least one comorbidity	45	
Arterial Hypertension	26	
Type 2 diabetes mellitus	7	
Dyslipidemia	13	
Obstruct sleep apnea	21	
Joint problems	23	
Depressive disorder	23	
Anthropometric data	Before surgery - After surgery	
Weight (kg)	121,05±22,56	80,1±17,25
BMI (kg/m2)	43,06±5,87	28,48±4,89
Overweight (kg)	51,01±18,48	10,01±14,08
Overweight (%)	40,93±7,66	9,72±15,25
Loss overweight (%)	85,46±23,6	

The complications reported by patients include: hair loss (n=1), dizziness (n=2), gastroesophageal reflux disease (n=1), hypotension (n=1), weakness (n=1), epigastric pain (n=1), and tremor (n=1). No patient reported more than one complication, and only one required reoperation, but did not justify the reason.

## DISCUSSION

Several studies document the influence of weight loss in improving the quality of life ^2^ ^,^ ^9^ ^,^ ^13^ ^,^ ^16^ ^,^ ^18^ ^,^ ^26^. This improvement was described by Hachem and Brennan^16^ in a systematic review finding that bariatric surgery produced better results compared with other treatments for obesity, especially in the first two years after surgery. In this study 83% of patients (n=39) were within that period. For Driscoll et al.^13^, however, long-term data have been inconsistent.

The average score of patients in the questionnaire of quality of life was 1.85±0.64, a maximum of three points. Similar data were obtained by Janik et al.^19^ which compares the technical approach of sleeve and bypass with Roux-en-Y and found no significant differences.

The BAROS' domains most highly evaluated by patients were "general condition" (average score of 0.92 up to 1.0), "working performance" (0.33/0.5) and "physical activity" (0.28/0.5). The areas "social activities" (0.2/0.5) and "sexual interest" (0.11/0.5) had the worst average. This happened because most patients have answered that there was no change in these two domains generating zero score. Other studies^2^ ^,^ ^18^, using the same methodology, found that questions related to the general state obtained the highest averages, while the sexual area resulted in lower averages.

Like other studies^18^, there was no significant relation (p>0.05) between BMI before and after surgery and the score obtained in BAROS. However, the EWL% was statistically significant (p<0.05) in the final score, diverging from the result of other analysis^18^.

A retrospective study of 407 patients published by Ortega et al.^24^ stated that younger people with lower BMI and higher abdominal circumference had higher rates in EWL%. In this series, in contrast, individuals with higher preoperative BMI obtained better results in the loss of excess weight (p<0.05).

Several studies show that the sleeve gastrectomy reduces mortality and the development of new comorbid conditions and worsening of already present diseases in obese individuals^10^ ^,^ ^28^. It should be noted that cardiovascular diseases have been considered as the most common causes of death around the world^30^, being intrinsically related to the effects of obesity, hypertension, dyslipidemia, obstructive sleep apnea and type 2 diabetes mellitus.

The prevalence of diabetes among the obese patients in the preoperative period was similar to the study of Blume et al.^4^ which showed a value of 14.7%. The full resolution of this disease was similar to other studies that have obtained rates of 47%^19^, 66%^14^ and up to 81%^12^. Hypertension is present between 45% and 68% of patients in the preoperative and presents clinical improvement or cure in up to 87% of patients in the postoperative^4^ ^,^ ^12^. The obstructive sleep apnea and dyslipidemia also are reduced after the procedure as demonstrated by Chang et al.^10^.

In addition to the above comorbidities, the high frequency of psychological diseases of obesity^15^ can lead to behavioral changes by the fact that these individuals are frequent targets of prejudice and discrimination^29^. Weight reduction and comorbidity was effective in decreasing depressive disorder in these patients. However, it should be understood that the emotional state can be changed by different causes, which also involve aspects related to self-esteem^1^.

Limitations of this study include the subjectivity of BAROS questionnaire, the non-equivalence of the samples in the postoperative period of time, not representativeness of the sample compared to the prevalence in the population. In addition, analysis of the quality of life would be more accurate if the assessment was carried out in different postoperative times of each patient, obtaining thus an evolutionary clinical parameter.

## CONCLUSION

Quality of life was affected by the reduction of excess weight and the metabolic changes with resolution of most of the evaluated comorbidities. The change of patient dissatisfaction regarding the ability to work, physical activity and general health were also considerable. The least significant results with respect to sexual and social activities interest are possibly related to self-assessment capacity. 
